# Prevalence of Myopia in Children and Adolescents: A Systematic Review of Malaysian Prevalence Studies

**DOI:** 10.21315/mjms-08-2025-592

**Published:** 2026-02-28

**Authors:** Eudocia Shu Yi Tan, Puneet Agarwal, Cheong Lieng Teng

**Affiliations:** 1 Ministry of Health Malaysia, Putrajaya, Malaysia; 2 Department of Ophthalmology, IMU University, Seremban, Negeri Sembilan, Malaysia; 3 Department of Family Medicine, IMU University, Seremban, Negeri Sembilan, Malaysia

**Keywords:** myopia, prevalence, Malaysia, children, adolescent

## Abstract

**Background:**

Myopia is a common eye problem that has increased in prevalence worldwide, including in Malaysia. This study aimed to determine the prevalence of myopia among children and adolescents aged 0 to 18 years in Malaysia and its associated sociodemographic factors.

**Methods:**

The preferred reporting items for systematic reviews and meta-analyses (PRISMA) guideline were used in this study. We searched PubMed and Scopus, supplemented by a targeted Google Scholar search, for studies published from 1990 to 2024 on the prevalence of myopia in Malaysia. Myopia was defined as a refractive error ≥ −0.50 D.

**Results:**

The meta-analysis included 14 quality-assessed studies from seven states in Malaysia, covering 13,367 children and adolescents. The overall pooled prevalence of childhood myopia from 1990 to 2024 was 17.18% (95% CI: 9.98, 25.86). The prevalence of myopia was notably lower among children aged 0 to 6 years at 2.54% (95% CI: 0.66, 5.60) than among children aged 7 to 12 years at 26.48% (95% CI: 17.29, 36.84) and adolescents aged 13 to 18 years at 42.71% (95% CI: 26.80, 59.44). The prevalence of myopia was higher in females than in males (odds ratio: 1.20; 95% CI: 1.09, 1.32). The Chinese population had the highest prevalence of myopia at 44.62% (95% CI: 35.94, 53.47) compared with other ethnicities.

**Conclusion:**

Our findings indicate that at least four in 10 children and adolescents aged 13 to 18 years are likely to have myopia in Malaysia. Targeted clinical and public health interventions should prioritise high-risk groups, particularly females, individuals of Chinese ethnicity, and those aged 7 to 18 years.

## Introduction

Myopia is the most prevalent eye disease and the primary cause of visual impairment in children and adolescents worldwide, especially in Asia (1, 2). Most cases of myopia are associated with excessive axial elongation of the eye. Excessive eyeball elongation causes light from distant objects to focus in front of the retina, resulting in blurred distance vision. Approximately one-third of children and adolescents worldwide suffer from childhood myopia, with significant differences in frequency across demographic groups (3).

The risk of developing myopia is significantly influenced by both genetic and environmental factors. Children with one myopic parent have a twofold risk, which increases to fivefold with two myopic parents (4). Higher educational exposure, lack of outdoor activity, and prolonged near work are linked to increased prevalence of myopia (4, 5). The issue of near work is closely linked to the excessive screen time reported in a local survey of children (6). The increasing early exposure of children to digital devices at home and in schools has also been associated with the rising prevalence of myopia (7). Furthermore, myopia has been demonstrated to be possibly associated with poor academic performance in both local literature and systematic reviews (8, 9).

Myopia increases the risk of developing eye conditions later in life. Even with proper refractive correction, individuals with myopia, especially those with high myopia, remain at higher risk of developing sight-threatening diseases such as open-angle glaucoma, cataracts, retinal tears, and myopic maculopathy (10). Myopic macular degeneration and retinal detachment are two major pathologic events that can result from severe myopia and cause irreversible vision loss (10).

Several prevalence studies on myopia have been conducted among children and adolescents of various age groups and ethnicities over the last few decades. In the absence of a population-based survey on myopia, a systematic review of the existing literature can help to summarise the estimated prevalence of myopia and identify its sociodemographic characteristics and temporal trends among children and adolescents in Malaysia.

## Methods

This systematic review was conducted following the PRISMA guidelines (11). The study protocol was registered in the International Platform of Registered Systematic Review and Meta-Analysis Protocols on 5 March 2025 (12).

### Search Strategy

We searched two bibliographic databases (PubMed and Scopus) and performed a target Google Scholar search. A bibliographic search was conducted using combinations of the following terms: “Adolescent,” “Child,” “Malaysia,” “Myopia,” and “Prevalence.” All relevant publications up to 31 December 2024 were considered for inclusion.

### Definitions

Children and adolescents were stratified into three groups: 0 to 6 years, 7 to 12 years, and 13 to 18 years. Myopia was defined as the spherical equivalent of refractive error of at least 0.50 diopters (D).

### Inclusion Criteria

- Prevalence studies in Malaysia
- Participants were children and adolescents aged 0 to 18 years
- Study design included cross-sectional studies
- Myopia was measured using validated methods, such as retinoscopy and refraction (subjective or using an autorefractor)

### Exclusion Criteria

- Retrospective studies from a clinical sample
- Myopia measurement methods not using validated methods (e.g., Snellen chart alone)

### Data Management

References were processed using the citation manager EndNote 20 (Philadelphia, PA: Clarivate Analytics; 2025). Relevant data (number of participants with and without myopia, sociodemographic variables such as age groups, gender, and ethnicity) from the included studies were independently extracted by a pair of investigators to minimise potential bias and error and resolve any disagreements.

### Quality Assessment

Methodological validity of the identified studies was assessed using the JBI critical appraisal checklist for prevalence studies (13).

### Data Synthesis

The meta-analysis was performed using MedCalc Statistical Software version 23.2.6 (Ostend, Belgium: MedCalc Software Ltd; 2025). Pooled prevalence rates were reported using fixed effects if the I^2^ is ≤ 50% or random effects if the I^2^ exceed 50%. Subgroup analysis of myopia prevalence at the age, gender, and ethnicity level was generated.

## Results

### Characteristics of the Included Studies

**Figure 1:**
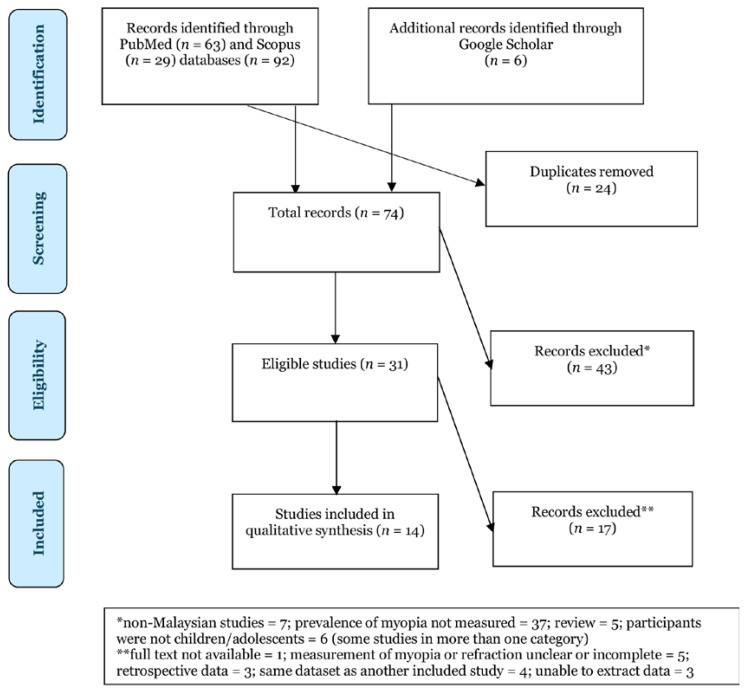
PRISMA flow chart

A total of 14 studies (number of participants = 13,367) published between 1990 and 2024 were included in this review (Figure 1 and Table 1). The study sites were mostly kindergartens and schools, with the exception of one community-based survey by Goh et al. (14) and one study conducted in a health clinic by Yahya et al. (15). All studies were based on samples taken from one or at most two states or provinces; no study had a nationally representative sample. Seven of the 14 studies were conducted in the Federal Territory, including Kuala Lumpur. Only one study by Premsenthil et al. (16) was conducted in Sarawak, and none were conducted in Sabah.

### Prevalence of Myopia According to Age, Gender, and Ethnicity

The overall prevalence of myopia was 17.18% (random effect model; 95% CI: 9.98, 25.86). As shown in Table 2, the prevalence of myopia increased with age: 0 to 6 years (five studies): 2.54% (95% CI: 0.66, 5.60); 7 to 12 years (eight studies): 26.48% (95% CI: 17.29, 36.84) and 13 to 18 years (three studies): 42.71% (95% CI: 26.80, 59.44).

Data for high myopia were reported in only three studies [Bakar et al. (17), Mohidin et al. (24) and Omar et al. (26)] with a reported prevalence of 1.75%, 0.40%, and 4.88%, respectively. The meta-analysis was not conducted because they used different definitions of high myopia.

**Figure 2:**
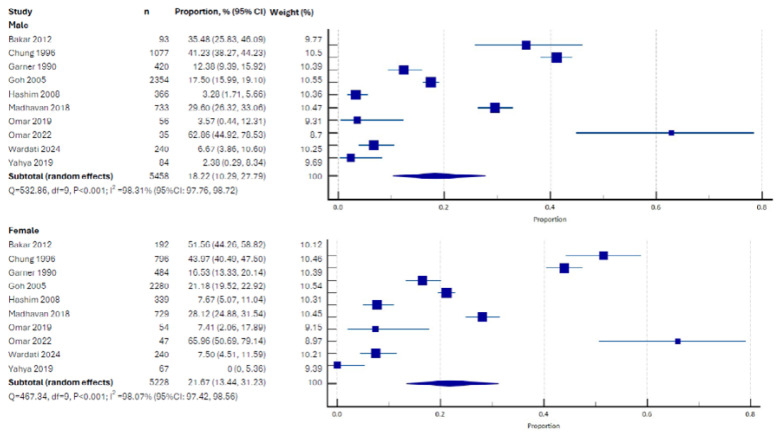
Comparison of myopia prevalence in female vs. male (proportion)

**Figure 3:**
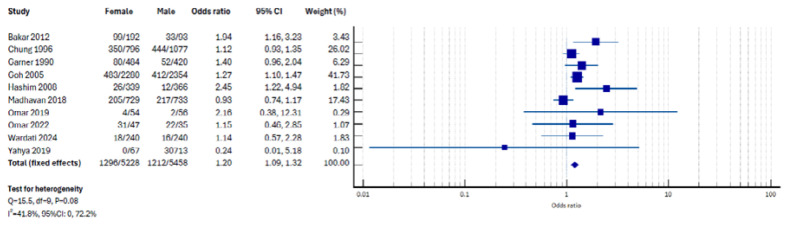
Comparison of myopia prevalence in female vs. male (odds ratio)

In 10 studies, gender data were extractable. The overall pooled prevalence of myopia was higher among females at 21.67% (95% CI: 13.44, 31.23), compared with 18.22% (95% CI: 10.29, 27.79) among males (Figure 2). The prevalence of myopia in females was higher than that in males, with an odds ratio of 1.20; 95% CI: 1.09, 1.32, *P* < 0.001 (Figure 3).

Ethnicity was categorised into four groups: Malay, Chinese, Indian, and others (primarily Orang Asli and Iban). Only seven studies recruited study participants from multiple ethnic groups (seven studies were single-ethnic studies); only one study recruited only Orang Asli, and another one from Sarawak that included some Sarawakian native populations. The overall pooled prevalence of myopia was the highest among Chinese (four studies) at 44.62% (95% CI: 35.94, 53.47), followed by Indian (four studies) at 17.55% (95% CI: 10.26, 26.30), others (five studies) at 17.40% (95% CI: 4.21, 37.00), and Malay (five studies) at 12.64% (95% CI: 5.55, 22.09) (Table 3). Comparison between ethnic groups cannot be assessed due to the insufficient number of studies.

Subgroup analysis at the urban or rural sample level could not be performed due to missing data. We did not analyse the temporal trend of myopia prevalence (by publication year) due to the small number of studies in each age group (at least 10 studies are needed for such analysis).

## Discussion

### Overall Prevalence

This meta-analysis synthesised data on the prevalence of myopia among children and adolescents in Malaysia from 14 studies published between 1990 and 2024. The overall pooled prevalence of myopia was 17.18% among children and adolescents in Malaysia. The overall prevalence may not be very useful due to high heterogeneity in view of variabilities in participant characteristics, especially age group, ethnicity, and methods of myopia detection.

### Sociodemographic Variables of Prevalence of Myopia

Subgroup analysis by age groups showed increasing myopia prevalence by age from 2.54% among those 0 to 6 years, to 26.48% among those 7 to 12 years and to 42.71% among those 13 to 18 years. The dramatic increase in the prevalence of myopia in the teenage years is consistent with other systematic reviews of prevalence studies (28, 29). One of the most widely recognised explanations for the development of myopia with age is a disproportionate increase in the axial length of the eyeball throughout the ocular development phase, usually during childhood (30).

Our systematic review found that females had a statistically significantly higher prevalence of myopia in children and adolescents: female vs. male odds ratio 1.20 (95% CI: 1.09, 1.32, *P* < 0.001). This finding is also noted in other systematic reviews from China, the Eastern Mediterranean Region, and globally (28, 29,31), but this gender difference was not seen in reviews of African and European studies (32, 33). Some researchers have proposed that the slight preponderance of myopia among females may be attributed to the earlier onset of puberty, which is linked to earlier eye growth and axial length elongation, hence contributing to myopia (34).

In this systematic review, the prevalence of myopia in the Chinese appears to be higher (44.62% vs. 12.64 to 17.55% in non-Chinese ethnic groups). Due to limitations in the included studies, we could not directly compare the prevalence by ethnic groups. Among the 14 included studies, only Goh et al. (14) reported a high prevalence of myopia among Chinese compared with all other ethnic groups. Similarly, Karuppiah et al. (35) consistently showed that Chinese primary and secondary school students in Singapore had a much higher prevalence of myopia than non-Chinese students. Systematic reviews have shown a high prevalence of myopia in several East Asian countries that share genetic and cultural affinity (3, 28), and the high prevalence has been attributed to cultural influences, especially the Confucian emphasis on education and filial piety. These characteristics encourage intense academic focus and lifestyle behaviours that might lead to myopia development in school-aged children and adolescents (36).

None of the 14 included studies reported the sensitivity/specificity of visual acuity test vs. myopia as detected using refraction. If the diagnostic performance of visual acuity testing using screening methods, such as the Snellen chart or Early Treatment Diabetic Retinopathy Study (ETDRS) charts, is sufficiently high, this could support the use of these simpler tools in the school-based vision screening programme. In the study by Tong et al. (37), the sensitivity and specificity of visual acuity screening (at logMAR 0.28 or Snellen chart 6/11.5) vs. refraction had a sensitivity of 72%, specificity of 97%, positive predictive value of 96%, and negative predictive value of 78%.

### The Strengths and Limitations of the Review

This review has several limitations. First, the temporal trend of myopia prevalence among children and adolescents in Malaysia was not assessed due to the limited number of eligible studies over time. Second, the review did not comprehensively explore the various risk factors influencing the epidemiology of myopia in this population, as many included studies lacked sufficient detail or reporting consistency. Additionally, only a subset of relevant studies could be included in the meta-analysis due to incomplete data, which may limit the pooled estimates’ generalizability. Most studies employed a two-stage assessment for detecting myopia, typically beginning with visual acuity screening using a Snellen chart or equivalent methods, followed by refraction testing. Individuals with visual acuity within a certain threshold are often assumed to have no refractive error, which may lead to an underestimation of the prevalence of true myopia. Furthermore, some studies have used noncycloplegic autorefraction, which can result in overestimation, particularly in younger children, due to accommodative responses (38). These methodological variations may contribute to inconsistencies in reported prevalence rates and highlight the need for standardised diagnostic approaches, such as using only cycloplegic refraction to diagnose myopia.

The high heterogeneity found in this systematic review points to considerable variability among studies, e.g., patient characteristics (age, gender, and ethnic differences), changing prevalence of myopia over time, and variable myopia detection methods. However, Migliavaca et al. (39) noted that prevalence meta-analyses often present high I^2^ values, which are not always synonymous with high heterogeneity.

Despite the above limitations, a key strength of this review is the rigorous selection of studies included in the analysis. Myopia measured using non-validated methods was excluded. Moreover, clinic-based studies were excluded because their recruited patients were less likely to be typical healthy children and adolescents. This enhances the reliability and validity of the pooled prevalence estimates and overall findings presented in this meta-analysis.

### Implications of the Study

This review of myopia prevalence in Malaysia revealed a relatively high prevalence, especially among Malaysian adolescents, more so among females than males, and especially among Chinese compared to other ethnicities. Given the potential adverse impact of myopia on educational attainment, early detection of myopia among children in rural areas and marginalised communities is critical. Myopia screening in Malaysia has been part of the School Health Services of the Ministry of Health Malaysia since 1967 (40). However, there are still significant gaps in the provision of vision screening and myopia correction. To date, we have not been able to find a comprehensive report on the Malaysian school-based vision screening programme. If the high prevalence in specific Malaysian communities is confirmed, there is a strong case to initiate intervention to reduce its prevalence, possibly targeting the known modifiable risk factors such as increased near work, high screen time, and inadequate outdoor activities (41).

## Conclusion

This systematic review and meta-analysis summarised the prevalence of myopia among children and adolescents in Malaysia, including data on sociodemographic subgroups. More comprehensive reporting of the prevalence of myopia, perhaps using detection methods easily used by nurses, of school-going children with follow-up data on myopia correction.

## Figures and Tables

**Table 1:** Characteristics of studies reporting the prevalence of myopia among school-going children and adolescents in Malaysia

Study	Study site	*N*	Demographic data	Visual acuity screening	Cycloplegia refraction assessment	Prevalence of myopia
Goh et al. 2005 (14)	Kuala Lumpur, schools and community	4,634	Age: 7 to 15 y Ethnicity: Malay 70.3%, Chinese 16.5%, Indian 8.9%, Others 4.3% Gender: Male 50.8%, female 49.2%	Tumbling-E Optotypes < 20/40	Cycloplegia: Yes Retinoscopy and autorefraction	19.3% with retinoscopy 20.7% with autorefraction
Yahya et al. 2019 (15)	Kuala Lumpur, health clinic	151	Age: 0.5 to 3 y Ethnicity: Malay 83.4%, Chinese 6.6%, Indian 9.3%, others 0.7% Gender: Male 55.6%, female 44.4%	None	Cycloplegia: Yes Retinoscopy and refraction	1.3%
Premsenthil et al. 2013 (16)	Sarawak, kindergartens	400	Age: 4 to 6 y Ethnicity: Malay 28.8%, Chinese 32.8%, Iban 15%, Bidayuh 19%, Others 4.5% Gender: Male 49%, female 51%	Sheridan Gardiner chart and Cardiff charts ≤ 6/12	Cycloplegia: Yes Refraction	3.75%
Bakar et al. 2012 (17)	Sarawak, schools	285	Age: 7, 12, 15 y Ethnicity: Malay 59.6%, Iban 40.4% Gender: Male 32.6%; female 67.4%	Snellen chart ≤ 6/9	Cycloplegia: Yes Retinoscopy and subjective refraction	46.3%
Chew et al. 2018 (18)	Johor, kindergartens	1,287	Age: 4 to 6 y Ethnicity: Malay 54.8%, Chinese 27.7%, Indian 15.6%, OA 1.9% Gender: Male 52.3%, female 47.7%	LogMAR EDTRS chart ≤ 0.2	Cycloplegia: Yes Retinoscopy	9%
Chung et al. 1996 (19)	Kuala Lumpur, schools	1,873	Age: 6 to 18 y Ethnicity: Chinese 100% Gender: Male 57.5%, female 42.5%	Snellen chart ≤ 6/9	Cycloplegia: No Retinoscopy	42.4%
Garner et al. 1990 (20)	Kuala Lumpur, schools	904	Age: 6 to 17 y Ethnicity: Malay 100% Gender: Male 46.5%, female 53.5%	Snellen chart NA	Cycloplegia: No Retinoscopy	8% (7 to 12 y), 20% (13 to 17 y)
Hashim et al. 2008 (21)	Kelantan, schools	705	Age: 6 to 12 y Ethnicity: Malay 100% Gender: Male 51.9%, female 48.1%	LogMAR ETDRS chart < 20/40	Cycloplegia: No Retinoscopy and autorefraction	5.4%
Ismail et al. 2022 (22)	Kuala Lumpur, schools	245	Age: 8 to 12 y Ethnicity: NA Gender: Male 41.2%, female 58.8%	Snellen chart ≤ 6/9	Cycloplegia: Yes Retinoscopy and autorefraction	30.2%
Madhavan et al. 2018 (23)	Kuala Lumpur, schools	1,462	Age: 7 to 11 y Ethnicity: Indian 100% Gender: Male 50.1%, female 49.9%	LogMAR EDTRS chart < 0.3	Cycloplegia: No Autorefraction	28.9%
Mohidin et al. 2005 (24)	Kuala Lumpur, schools	749	Age: 7 to 18 y Ethnicity: Indian 100% Gender: Male 50%, female 50%	NA	Cycloplegia: No Retinoscopy	17.6%
Omar et al. 2019 (25)	Negeri Sembilan, schools	110	Age: 7 to 12 y Ethnicity: OA 100% Gender: Male 50.9%, female 49.1%	LogMAR EDTRS chart < 0.3	Cycloplegia: Yes Refraction	5.5%
Omar et al. 2022 (26)	Pahang, schools	82	Age: 7 to 12 y Ethnicity: Chinese 100% Gender: Male 42.7%, female 57.3%	Snellen chart NA	Cycloplegia: NA Refraction	64.6%
Wardati et al. 2024 (27)	Terengganu and Pahang, schools	480	Age: 4 to 6 y Ethnicity: Malay 99.4%, OA 0.6% Gender: Male 50%, female 50%	Snellen chart NA	Cycloplegia: No Refraction	7.1%

N = sample size; NA = not available; OA = Orang Asli; y = year

* The study quality of included studies varies between 6 and 8; reasons for lower for some studies were: small sample size, convenient sample, absence of response rate

**Table 2:** Prevalence of myopia by age groups

Study	Sample size	Proportion, % (95% CI)	Weight (%)
**Age group 0 to 6 y**			

Yahya et al. 2019 (15)	151	1.33 (0.16, 4.70)	19.06
Premsenthil et al. 2013 (16)	400	3.75 (2.11, 6.11)	21.67
Chew et al. 2018 (18)	1,287	1.01 (0.54, 1.72)	22.99
Hashim et al. 2008 (21)	55	0 (0, 6.49)	14.31
Wardati et al. 2024 (27)	480	7.08 (4.96, 9.76)	21.97
Subtotal (random effects)	2,373	2.54 (0.66, 5.60)	100.00
Q = 45.23, *df* = 4, *P* < 0.001; I^2^ = 91.16% (95%CI: 82.32, 95.57)			

**Age group 7 to 12 y**			

Goh et al. 2005 (14)	3,461	16.09 (14.88, 17.36)	12.99
Bakar et al. 2012 (17)	163	42.95 (35.23, 50.92)	12.27
Chung et al. 1996 (19)	1,049	36.61 (33.69, 39.60)	12.90
Hashim et al. 2008 (21)	650	5.85 (4.17, 7.94)	12.83
Ismail et al. 2022 (22)	245	30.20 (24.52, 36.37)	12.51
Madhavan et al. 2018 (23)	1,462	28.87 (26.55, 31.26)	12.94
Omar et al. 2019 (25)	110	5.46 (2.03, 11.50)	11.94
Omar et al. 2022 (26)	82	64.63 (53.30, 74.88)	11.62
Subtotal (random effects)	7,222	26.48 (17.29, 36.84)	100.00
Q = 510.41, *df* = 7, *P* < 0.001; I^2^ = 98.63% (95%CI: 98.16, 98.98)			

**Age group 13 to 18 y**			

Goh et al. 2005 (14)	1,173	28.82 (26.24, 31.50)	34.27
Bakar et al. 2012 (17)	122	50.82 (41.62, 59.98)	31.60
Chung et al. 1996 (19)	824	49.76 (46.29, 53.23)	34.13
Subtotal (random effects)	2,119	42.71 (26.80, 59.44)	100.00
Q = 99.30, *df* = 2, *P* < 0.001; I^2^ = 97.99% (95%CI: 96.26, 98.92)			

**All studies**			

Total (random effects)	13,367	17.18 (9.98, 25.86)	100.00
Q = 1788.38, *df* = 13, *P* < 0.001; I^2^ = 99.27% (95%CI: 99.13, 99.39)			

**Table 3:** Prevalence of myopia by ethnicity

Study	Sample size	Proportion, % (95% CI)	Weight (%)
**Malay**			

Bakar et al. 2012 (17)	115	52.17 (42.66, 61.58)	18.92
Goh et al. 2005 (14)	3,257	13.88 (12.71, 15.11)	20.92
Hashim et al. 2008 (21)	705	5.39 (3.84, 7.32)	20.63
Wardati et al. 2024 (27)	477	7.13 (4.99, 9.82)	20.45
Yahya et al. 2019 (15)	126	1.59 (0.19, 5.62)	19.08
Total (random effects)	4,680	12.64 (5.55, 22.09)	100.00
Q=180.94, df=4, *P*<0.001; I^2^ = 97.79% (95%CI: 96.51, 98.60)			

**Chinese**			

Chung et al. 1996 (19)	1,873	42.39 (40.14, 44.67)	35.20
Goh et al. 2005 (14)	764	45.29 (41.72, 48.90)	34.07
Omar et al. 2022 (26)	82	64.63 (53.30, 74.88)	23.54
Yahya et al. 2019 (15)	10	0 (0, 30.85)	7.20
Total (random effects)	2,729	44.62 (35.94, 53.47)	100.00
Q=30.96, df=3, *P*<0.001; I^2^ = 90.31% (95%CI: 78.17, 95.70)			

**Indian**			

Goh et al. 2005 (14)	412	15.53 (12.17, 19.40)	28.90
Madhavan et al. 2018 (23)	1,462	28.87 (26.55, 31.26)	30.20
Mohidin 2005	749	17.62 (14.96 to 20.55)	29.70
Yahya et al. 2019 (15)	14	0 (0, 23.16)	11.20
Total (random effects)	2,637	17.55 (10.26, 26.30)	100.00
Q=62.36, df=3, *P*<0.001; I^2^ = 95.19% (95%CI: 90.64, 97.53)			

**Others**			

Bakar et al. 2012 (17)	170	42.35 (34.82, 50.15)	26.94
Goh et al. 2005 (14)	201	16.42 (11.58, 22.28)	27.07
Omar et al. 2019 (25)	110	5.46 (2.03, 11.50)	26.51
Wardati et al. 2024 (27)	3	0 (0, 70.76)	11.91
Yahya et al. 2019 (15)	1	0 (0, 97.50)	7.58
Total (random effects)	485	17.40 (4.21, 37.00)	100.00
Q = 64.36, *df* = 4, *P* < 0.001; I^2^ = 93.78% (95%CI: 88.38, 96.68)			
